# The Trend of Arrhythmias in Patients With COVID-19: A Complication or Late Manifestation?

**DOI:** 10.7759/cureus.50746

**Published:** 2023-12-18

**Authors:** Yusuf A Siddique, Raheel Chaudhry, Muhammad Ahmad, Ahmad Sebai, Lubhani Sharma, Mohamed Hassouba, Ghazala S Virk

**Affiliations:** 1 Basic Sciences, St. George's University School of Medicine, True Blue, GRD; 2 Medicine, Baylor College of Medicine, Houston, USA; 3 Medicine, Bolan Medical College, Quetta, PAK; 4 School of Medicine, California University of Science and Medicine, Colton, USA; 5 Family Medicine, Dayanand Medical College and Hospital, Ludhiana, IND; 6 Pediatrics, SUNY Downstate Health Sciences University, Brooklyn, USA; 7 Internal Medicine, Avalon University School of Medicine, Ohio, USA

**Keywords:** type 2 myocardial infarction, thromboinflammation, cytokine storm, endothelial damage, cardiovascular disease, ace2, covid-19

## Abstract

Patients diagnosed with coronavirus disease (CVD) who experience cardiovascular complications or have pre-existing cardiovascular disease are at an increased risk of death. The primary heart-related consequences associated with COVID-19 encompass venous thromboembolism, shock, heart failure, arrhythmias, myocarditis, acute myocardial infarction, and acute cardiac damage. The coronavirus has the potential to induce cardiovascular complications or exacerbate pre-existing CVD through various mechanisms. These mechanisms include dysregulation of the renin-angiotensin-aldosterone system; direct viral toxicity; damage to endothelial cells; formation of blood clots and subsequent inflammation, a phenomenon known as thromboinflammation; an excessive immune response known as cytokine storm; and an imbalance between the demand and supply of oxygen in the body. In this study, we comprehensively analyze the cardiovascular symptoms, histology, and underlying mechanisms associated with COVID-19. Our aim is to contribute to the identification of future research objectives and aid in the advancement of therapeutic management approaches.

## Introduction and background

The COVID-19 pandemic has been attributed to the transmission of severe acute respiratory syndrome coronavirus 2 (SARS-CoV-2), leading to a global infection count of more than 20.9 million individuals and a total of 760,633 fatalities as of August 15, 2020 [1]. SARS-CoV-2 belongs to the same viral family as the coronaviruses responsible for SARS and Middle East respiratory syndrome (MERS) [2]. In contrast to MERS and SARS, COVID-19 has lower death rates and more transmissibility, but its mortality rate remains higher than that of influenza [3]. The reported death rate of COVID-19 was approximately 3.4% for all patients, 1.4% for individuals without underlying diseases, and 13.2% for individuals who already have cardiovascular diseases (CVDs) [4]. The frequency of cardiovascular comorbidities among individuals hospitalized with COVID-19 has been shown to vary between 17.1% and 59.6%. Furthermore, the death rate among those with pre-existing CVDs or hypertension is 1.42 times and 3.15 times higher, respectively, compared to individuals without these conditions [5-9]. The escalating mortality rates associated with COVID-19, particularly among individuals with pre-existing medical conditions, have prompted significant interest in the cardiovascular implications of this viral infection.

The COVID-19 pandemic has the potential to lead to cardiovascular problems or exacerbate pre-existing CVD through both direct and indirect routes. Furthermore, there exists a positive correlation between the severity of COVID-19 and the presence of cardiovascular symptoms. In light of the ongoing global expansion of the COVID-19 pandemic, there has been a growing need for a more comprehensive comprehension of the intricate relationship between viral infection and the cardiovascular system. To meet this need, we present an extensive and up-to-date analysis of the cardiovascular presentations and underlying mechanisms of COVID-19. This article seeks to enhance our comprehension of the probable processes that underlie the impacts of COVID-19 on the cardiovascular system by integrating pathological and clinical results. The ultimate goal is to establish a basis for developing more effective preventative and therapeutic management approaches. Furthermore, the study aims to contribute to the identification of future research targets regarding COVID-19 and to help develop therapeutic management approaches.

Research question

The study's main purpose is to analyze and gain in-depth knowledge of cardiovascular systems and their underlying mechanisms associated with COVID-19. The study investigates where COVID-19 gives rise to cardiovascular complications or worsens pre-existing cardiovascular complications and will explore the mechanism by which that occurs.

Main claims

1. Patients who have been diagnosed with COVID-19 and have cardiovascular diseases and pre-existing diseases are at risk and have more chances of death.

2. Those who are diagnosed with COVID-19 have cardiovascular complications, including myocarditis, acute myocardial infarction, heart failure, venous thromboembolism, shock, and acute cardiac damage.

3. The mechanisms leading to cardiovascular symptoms of COVID-19 include dysregulation of the renin-angiotensin-aldosterone system, direct viral toxicity, endothelial cell damage, blood clot formation, and subsequent inflammation (thrombotic inflammation), cytokines, and an imbalance in oxygen demand.

4. Acute heart damage is common in COVID-19 patients and is associated with a higher risk of death.

5. COVID-19 may be associated with acute myocardial infarction, possibly due to endothelial dysfunction and a hypercoagulable state.

6. Myocarditis in COVID-19 patients may be more influenced by immune-mediated hyperinflammation than direct viral invasion.

7. There is a significant relationship between the severity of COVID-19 and the occurrence of various cardiac arrhythmias.

8. Heart failure, shock, and venous thromboembolism are the most common cardiovascular symptoms in COVID-19 patients.

9. ACE2-dependent viral toxicity leading to SARS-CoV-2 entry into cells may contribute to COVID-19-related cardiovascular injury and potentially increase disease susceptibility in individuals with a history of cardiovascular disease.

How this research fits within existing literature

The results of this study are consistent with the existing literature on the cardiovascular consequences of COVID-19. It deepens the understanding of how the virus affects the cardiovascular system and highlights a variety of symptoms, such as acute cardiac injury, myocardial infarction, cardiac arrhythmias, heart failure, and thromboembolic events. The study contributes to the broader debate about the complex relationship between COVID-19 and cardiovascular disease and highlights the need for further research and improved management strategies.

## Review

Cardiovascular manifestations

The clinical cardiovascular presentations of COVID-19 predominantly encompass acute myocardial infarction (AMI), pulmonary embolism (PE), shock, acute cardiac injury (ACI), heart failure, arrhythmia, myocarditis, and venous thromboembolism (VTE).

ACI

ACI is characterized by an elevation in cardiac troponin levels, accompanied by potential decreases in the ejection fraction or the presence of electrocardiographic abnormalities. The occurrence rate of acute cardiac damage in individuals diagnosed with COVID-19 ranged from 10% to 23% [10-13]. Notably, patients admitted to the intensive care unit (ICU) exhibited a greater prevalence of ACI compared to non-ICU patients (22.2% vs. 2.0%) [13]. Furthermore, a significantly higher proportion of non-survivors experienced ACI compared to survivors (59.0% versus 1.0%) [12]. A study also found that individuals experiencing ACI exhibited more pronounced illness manifestations, such as creatinine, N-terminal pro-B-type natriuretic peptide (NT-proBNP), and elevated levels of C-reactive protein [14]. In addition, these patients displayed a greater prevalence of ground-glass opacity and multiple mottling on imaging scans and were more inclined to receive either invasive or noninvasive ventilation. Malignant arrhythmias and cardiac dysfunction were found to be related to acute cardiac damage [14]. This study also found that individuals who experienced ACI had a considerably elevated risk of death [15].

In another research, this risk was observed both throughout the period from the beginning of symptoms (hazard ratio (HR) = 4.26; 95% confidence interval (CI) = 1.92-9.49) and from admission to the endpoint (HR = 3.41; 95% CI = 1.62-7.16) [15]. A stronger degree and more frequent occurrence of an increase in cardiac troponin enzymes were also found to be correlated with increased mortality. Individuals diagnosed with COVID-19 who have a history of cardiovascular disease (CVD) exhibit a heightened susceptibility to experiencing acute cardiac damage [15]. In this research, in comparison to patients who did not experience cardiac injury, individuals with cardiac injury exhibited a significantly increased prevalence of CVD (29.3% vs. 6.0%) and hypertension (59.8% vs. 23.4%). Furthermore, individuals with CVD comorbidities who contract SARS-CoV-2 often have a decline in cardiac reserve and reduced ability to tolerate hypoxia. Consequently, they are at an increased risk of developing cardiac insufficiency, such as shock, malignant arrhythmia, and heart failure.

AMI

Respiratory viruses, such as severe acute respiratory syndrome (SARS) and influenza, have been found to be linked to AMI due to their ability to elevate the risk of rupture of plaque deposited in coronary blood flow. A study also found that the incidence ratio of AMI in individuals within the first week after being diagnosed with influenza is 6.05 (95% CI = 3.86-9.50) compared to one year after influenza diagnosis [16]. AMI has been observed in individuals diagnosed with COVID-19; however, the exact incidence of such occurrences remains undetermined. According to electrocardiographic research conducted on individuals with coronavirus, the occurrence of recently diagnosed AMI was documented in 5.3% of these cases [17]. In addition, another investigation utilizing echocardiography found a prevalence of 2.9% for newly diagnosed AMI [18]. The preliminary clinical presentation of the coronavirus can include ST-elevation myocardial infarction (STEMI), with a significant proportion of individuals (33.3-39.3%) identified with non-obstructive coronary artery disease [19,20]. This observation suggests a potential association between COVID-19 and endothelial dysfunction and a hypercoagulable condition.

The COVID-19 pandemic has significantly altered the trajectory of individuals with AMI. Hospitalization rates for AMI experienced a decline during the initial stages of the COVID-19 pandemic. However, there was a slight improvement in these rates after a period of five weeks, albeit accompanied by an increase in in-hospital mortality. This trend was observed in the United States [21]. During the COVID-19 pandemic, a considerable number of individuals experiencing AMI chose to forgo hospitalization, potentially due to concerns regarding the transmission of SARS-CoV-2. This decision resulted in a subsequent delay in seeking medical attention and exacerbation of the AMI condition. Additional research will be required to clarify the effects of COVID-19 on AMI.

Myocarditis

Myocarditis is characterized as a myocardial injury resulting from a direct viral invasion of the cardiac tissue. During the initial phase of the coronavirus pandemic, a study found that out of a total of 68 individuals suffering from myocardial damage, five individuals (7%) succumbed to circulatory failure [22]. In addition, another case study identified a patient with a third-degree atrioventricular block who was diagnosed with myocarditis [18]. Several cases of myocarditis connected to COVID-19 have been established through the utilization of cardiac magnetic resonance imaging (MRI) [23-26] However, the available evidence supporting viral entrance into heart cells is limited. The endomyocardial biopsy revealed the presence of lymphocytic inflammatory infiltrates in the myocardium. However, the detection of SARS-CoV-2 particles was limited to the interstitial cells of the myocardium [27]. A recent instance of coronavirus presented with lymphocytic myocarditis, wherein the myocardium did not exhibit the existence of coronavirus [28]. Therefore, it is plausible that immune-mediated hyperinflammation may exert a more substantial influence on the pathophysiology of acute myocarditis linked to COVID-19, surpassing the impact of viral replication or toxicity. There have been reports of pericardial involvement in cases of cardiac tamponade [29].

Arrhythmia

Research has proposed that there is a significant correlation between the severity of COVID-19 and the presence of diverse arrhythmias. This study was conducted on 138 COVID-19 patients who were admitted to the hospital and revealed that arrhythmias were observed in 16.7% of the cases [18]. Notably, a higher percentage of patients in the ICU (44.4%) experienced arrhythmias compared to those in non-ICU settings (6.9%) [18]. This difference was shown to be statistically significant (P < 0.001) [18]. In comparison to individuals in the non-ICU group, the percentage of aberrant Q waves observed in electroencephalography (ECG) recordings from individuals in the ICU group exhibited a substantial increase (33.3% versus 3.9%, P = 0.006) [18]. The prevalence of ventricular arrhythmias is much greater in individuals with ACI compared to those without ACI (17.3% vs. 1.5%, P < 0.001) [14]. Patients who required mechanical breathing exhibited a higher prevalence of atrial arrhythmias compared to those who did not (17.7% vs. 1.9%) [30]. A study conducted in New York observed that a significant proportion (6%) of the 4,250 patients diagnosed with COVID-19 exhibited a prolonged corrected QT interval above 500 ms. These findings highlight the need to not disregard this cardiac abnormality in individuals affected by the disease [23]. Among a cohort of 136 patients with COVID-19 who suffered from cardiac arrest while hospitalized, it was observed that asystole was the predominant initial rhythm in 89.7% of cases, while shockable rhythms were seen in a mere 5.9% of patients [31]. Instances of sudden cardiac death have been documented in individuals diagnosed with coronavirus who originally presented with modest symptoms but subsequently succumbed to the disease while in their residences [32].

In contrast to the decrease observed in the number of patients diagnosed with AMI during the initial stages of the COVID-19 pandemic, there was a notable rise of 58% in out-of-hospital cardiac arrests reported within the first 40 days of the COVID-19 outbreak as compared to the corresponding period in the previous year (2019). The occurrence may have been influenced by the combination of coronavirus or other untreated conditions, such as AMI, along with patients' reluctance to seek medical care [33]. Arrhythmias observed in individuals with COVID-19 are believed to arise as a result of many factors, including electrical instability in conjunction with adrenergic stress, AMI, myocarditis, hypoxemia, systemic inflammation, electrolyte imbalances, metabolic dysregulation, and the administration of medications that lengthen the QT interval.

Heart Failure

A study found that 23% of patients diagnosed with COVID-19 experienced heart failure. Furthermore, according to this research, the incidence of heart failure was much higher in patients who did not survive compared to those who did survive (52% vs. 12%, P < 0.001) [34]. Another research proposed that the elevation of B-type natriuretic peptide (BNP) and N-terminal pro-B-type natriuretic peptide (NT-proBNP) in patients with COVID-19 is associated with an unfavorable prognosis, as indicated by cardiac markers, such as troponin [22]. In the population of individuals afflicted with severe cases of coronavirus, it has been observed that a range of 7-33% experienced biventricular failure [35]. Previous research has also documented instances of right ventricular failure occurring independently or in conjunction with PE [36]. Several case studies have documented an emerging association between stress cardiomyopathy, also known as COVID-19 and Takotsubo syndrome. Then, the incidence of stress cardiomyopathy had a substantial rise (7.8%) during the COVID-19 pandemic in comparison to the rates observed before the outbreak (1.5-1.8%) [37]. The COVID-19 pandemic has given rise to various challenges, including mental, social, and financial burdens, which have been postulated to potentially trigger stress cardiomyopathy [38-40]. The pathophysiology of stress cardiomyopathy may involve the involvement of sympathetic stress cytokine storm and microvascular dysfunction.

Heart failure can potentially develop as a chronic outcome of cardiac infection caused by the SARS-CoV-2 virus, representing an advanced-stage manifestation of CVD. In a recent cohort research with a sample of 100 individuals who had recovered from coronavirus, the presence of cardiac involvement was observed in 78 individuals, while chronic myocardial inflammation was detected in 60 patients using cardiac MRI. These findings were independent of the severity of illness or the presence of pre-existing CVD (42).

Shock

Mixed, septic, and cardiogenic shock represents a significant criterion for identifying serious illness in individuals affected by COVID-19. In a research, shock occurred in 8.7% of the 138 patients diagnosed with COVID-19, and its incidence was higher among patients admitted to the ICU in comparison with those who were not (30.6% vs. 1.0%, P < 0.001) (10). Determining the presence of a concurrent cardiogenic factor is of utmost importance in aiding clinical decision-making, especially in cases where extracorporeal membranous oxygenation (ECMO) is necessary for mechanical respiratory and circulatory support. This assessment holds significance as it can potentially impact the choice of device, whether it be venovenous or venoarterial, for the aforementioned purpose [41].

During the latter stage of the COVID-19 pandemic, there has been a significant focus in the United States and Europe on healthy children who exhibit atypical symptoms resembling Kawasaki disease (KD) [42,43]. The syndrome is known as pediatric inflammatory multisystem syndrome temporally linked with SARS-CoV-2 (PIMS-TS). The clinical presentation of this condition was characterized by persistent fever, indications of inflammation (e.g., elevated levels of neutrophils, C-reactive protein, and reduced lymphocyte count), and the presence of dysfunction in one or multiple organs (including shock, neurological, respiratory, gastrointestinal, oval, and renal impairments). These manifestations were observed in individuals who had a concurrent or prior infection with the coronavirus. When comparing PIMS-TS with KD shock syndrome or KD, it was observed that higher levels of inflammatory markers, for example, C-reactive protein (CRP) and older age, were more prevalent [44]. In a subset of patients, approximately 25% of individuals with untreated KD may develop coronary aneurysms. The onset of PIMS-TS may be attributed to immunological dysregulation generated by SARS-CoV-2, as evidenced by the presence of antibodies against coronavirus in a significant proportion of children with PIMS-TS, despite the absence of nucleic acids [45].

VTE and PE

Coronavirus has been linked to thrombotic and proinflammatory disorders that have the potential to lead to thromboembolic events [46]. Indeed, there is a correlation between elevated indicators of thrombosis and more unfavorable clinical outcomes. The study conducted in multiple centers in China found that elevated levels of D-dimer (>1 g/l) were identified as independent predictors of mortality during hospitalization [12]. In addition, it is worth noting that a significant proportion of patients, specifically 71.4%, who experienced mortality were found to have been diagnosed with disseminated intravascular coagulation [47]. The study findings revealed a substantial prevalence of VTE among patients in the ICU, with a reported incidence rate of 69% (50). However, previous studies have documented a lower incidence of VTE at other medical centers, with a reported rate of 22.2% [48].

Despite the administration of preventative anticoagulation, a notable proportion of patients, specifically 31%, who suffered from COVID-19 experienced the development of VTE [49]. In addition, a considerable percentage of patients, specifically 16.7%, were diagnosed with PE [49]. The incidence of PE was shown to be higher in those with COVID-19 who also had acute respiratory distress syndrome (ARDS) compared to those without ARDS (11.7% vs. 2.1%, P < 0.008) [50]. A separate investigation documented a cumulative occurrence of PE at 20.4% (95% CI = 13.1-28.7%) among critically ill individuals with COVID-19, which was significantly greater than the incidence observed among patients admitted to the same ICU over the corresponding time frame in 2019 [51].

Mechanisms of cardiac manifestations in COVID-19

The cause of COVID-19 is attributed to a combination of direct and indirect mechanisms leading to pathological manifestations. Direct damage occurs as a result of the viral infection of specific target cells. Indirect injuries primarily arise due to immunological responses, inflammatory reactions, circulatory impairments, and hypoxic conditions.

Angiotensin-Converting Enzyme 2 (ACE2)-Mediated Viral Toxicity

The expression of angiotensin-converting enzyme 2 (ACE2) has been observed in several components of the vascular system, including vascular smooth muscle cells and migratory angiogenic cells. In addition, ACE2 has been found in different cell types within the heart, such as epicardial adipose cells, cardiomyocytes, cardiac fibroblasts, pericytes, and endothelial cells [52]. ACE2 serves as a receptor for viral entry, facilitating the binding of the spike (S) protein from both SARS-CoV-2 and SARS-CoV. Furthermore, the process of cell entrance requires the activation of the S subunit through the action of the cellular serine protease TMPRSS2 (transmembrane protease serine 2) or other proteases, such as Furin, elastase, trypsin, factor X, cathepsin B, and cathepsin L [53].

The heightened transmissibility of coronavirus may be attributed to its stronger affinity for ACE2 compared to SARS-CoV [54]. Moreover, it has been observed that individuals with a history of CVD have more severe symptoms of COVID-19. This correlation may be attributed to their elevated plasma concentrations of ACE2 [54]. However, in comparison to the lung, the human heart has a greater expression of ACE2 and a significantly lower level of TMPRSS2. The cardiac vulnerability to COVID-19 infection was somewhat reduced due to a decreased prevalence of ACE2+/TMPRSS2+ cells. There is evidence to suggest that Furin and cathepsin L, which are highly expressed in the human heart, could potentially enhance the vulnerability of the heart to COVID-19 infection [55].

Dysregulation of RAAS

The enzyme ACE2 facilitates the conversion of angiotensin II (Ang II) into Ang-(1-7), and the ACE2/Ang-(1-7)/Mas axis plays a crucial role in counteracting the negative effects of the renin-angiotensin-aldosterone system (RAAS), hence maintaining the body's physiological and pathologic balance. The cellular entry of COVID-19 is facilitated by the interaction between the extracellular domains of ACE2 and spike protein (S protein), resulting in the reduction of ACE2 expression on the cell surface. The activity of Ang-II/angiotensin 1 receptor (AT1R) is subsequently heightened, while the ACE2/Ang 1-7/Mas axis is compromised, resulting in a range of detrimental effects, such as vasoconstriction, aldosterone secretion, hypertrophy, vascular permeability, fibrosis, pro-inflammatory responses, increased production of reactive oxygen species and cardiac remodeling, development of multiple organ dysfunction syndrome (MODS), and gut dysbiosis in individuals affected by COVID-19 [52-56].

ACE2 has a multitude of protective actions in diverse organs and illnesses, and the presence of genetic ACE2 deficiency is linked to unfavorable outcomes. For example, in a study, the ACE2 deletion mice exhibited myocardial hypertrophy and interstitial fibrosis, as well as exacerbated cardiac failure [57]. The pathogenesis in mice infected with influenza virus H5N1 was significantly aggravated by ACE2 deficiency, while the severity of lung injury was alleviated by the inhibition of AT1R [58]. ACE2 has been found to have the ability to induce insulin secretion and reduce insulin resistance [59]. The expression of ACE2 was observed to be downregulated in the cardiac cells of both mice and people infected with SARS-CoV [60]. A positive link has been seen between increased levels of circulating Ang II in individuals with COVID-19 and the occurrence of lung damage and/or viral load. In summary, there exists a partly causal relationship between the downregulation of tissue ACE2 and the overexpression of Ang II, which contributes to the emergence of multiorgan failure or cardiovascular problems subsequent to COVID-19-2 infection [61,62].

Thromboinflammation and Endothelial Cell Damage

The pathogenic mechanisms of the coronavirus involve both the direct attack of endothelial cells by coronavirus infection and the indirect production of prothrombotic and inflammatory conditions in vasculopathy [63,64]. It has been observed that both venous and arterial endothelial cells express ACE2 [64]. In addition, histological investigations have yielded microscopic observations of COVID-19 particles within endothelial cells of the kidney. These studies have also revealed the presence of endothelitis, characterized by the activation of macrophages and neutrophils, in other organs, such as the heart, gut, and lung [63]. A research also proposed that Von Willebrand factor (VWF), a glycoprotein involved in blood coagulation that circulates in the bloodstream, exhibits a notable increase in coronavirus patients when compared to persons without the disease [65]. VWF, which serves as a carrier for coagulation factor VIII, has the ability to initiate platelet aggregation and promote blood coagulation [66]. The interaction between neutrophils and platelets, direct invasion of endothelial cells by coronavirus, and indirect generation of prothrombotic and inflammatory conditions in vasculopathy both result in the pathophysiological mechanisms of COVID-19 [63-66]. It has been found that both arterial and vascular endothelial cells exhibit the expression of ACE2 [64].

Additionally, histological investigations have demonstrated microscopic evidence of coronavirus particles in kidney endothelial cells in addition to clear endothelitis in several organs, such as the heart, gut, and lung, which is characterized by activated macrophages and neutrophils [63]. VWF is a coagulation glycoprotein that circulates in the blood. It causes endothelial dysfunction significantly in individuals with COVID-19 compared to normal individuals [65]. VWF also carries factor VIII, which can elicit platelet aggregation in addition to blood coagulation [66]. After the platelet-neutrophil interaction, macrophage initiation can lead to proinflammatory reactions, including cytokine storm and the formation of neutrophil extracellular traps (NETs) [67].

The extrinsic and intrinsic coagulating pathways activate after the endothelial gets damaged by NETs. Due to this activation of coagulation pathways, the formation of microthrombus happens and further causes microvascular dysfunction. Elevated levels of NETs were observed in hospitalized COVID-19 patients and were positively correlated with disease severity [68]. Inhibiting NETs could be a therapeutic target for reducing NET-mediated thrombotic tissue damage in COVID-19 [69], followed by the activation of macrophages, which might contribute to the enhancement of proinflammatory reactions, such as the generation of NETs and cytokine storm. Neuroendocrine tumors have been found to cause harm to the endothelium, leading to the activation of both extrinsic and intrinsic coagulation pathways. This activation subsequently leads to the production of microthrombi and malfunction in the microvasculature. Elevated levels of NETs were seen in individuals who were admitted to the hospital because of COVID-19, and there was a positive association between the levels of NETs and the severity of the disease [68]. The inhibition of NETs could potentially serve as a therapeutic strategy for mitigating the thrombotic tissue damage caused by COVID-19, as suggested by recent research [69].

Cytokine Storm Triggered by Immune Dysregulation

The severe form of COVID-19 is distinguished by a dysregulated immune response and subsequent cytokine storm. Prior research involving human coronaviruses has documented that hyperinflammation is mediated by inflammatory cell recruitment (macrophages, neutrophils, and monocytes), interference with interferon signaling, and rapid viral replication [70]. ACI in individuals with coronavirus was found to be independently associated with inflammation parameters (procalcitonin and CRP) and neutrophils, lymphocytes, and white blood cells. A significant contributor to mortality, subsequent cytokine storm is distinguished by an abrupt increase in the concentration of numerous proinflammatory cytokines that are activated by the infection. It has been documented that infection with SARS [70,71], MERS, and H1N1 [70,71] results in a subsequent cytokine storm. An extensive analysis of the transcriptional response to COVID-19- 2 infection revealed an unconventional inflammatory response marked by elevated concentrations of interleukin (IL)-6, decreased levels of type I and III interferons, and increased chemokines. COVID-19 pathology is exacerbated by increased proinflammatory responses and diminished innate antiviral defenses [72].

 Zhou et al. [73] documented the inflammatory CD14+CD16+ monocyte-induced increased levels of IL-6 expression and granulocyte-macrophage colony-stimulating factor (GM-CSF) and pathogenic T helper 1 cells subsequent to COVID-19-2 infection. Serum IL-6 concentrations that are elevated have also been associated with an unfavorable prognosis [74,75] and were discovered to correlate with fibrinogen levels in COVID-19 patients [76]. IL-6 is capable of inducing thrombosis, activating coagulation, and inhibiting cardiac function [77,78], as well as causing endothelial dysfunction, which results in hypoxia, tissue ischemia, and vascular leakage; consequently, these conditions contribute to hypoxia, MODS, and disseminated intravascular coagulation.

The discrepancy between the supply and demand of oxygen

The primary presentation of COVID-19 is hypoxemia, which leads to inadequate oxygen delivery to organs that require high levels of energy and oxygen, including the heart [79]. The CI observed in coronavirus patients is believed to be produced by an imbalance between the demand and supply of oxygen, which is attributed to both cytokine storm and endothelial dysfunction. This mechanism is analogous to the pathophysiology observed in type 2 MI, where there is no abrupt breakdown of atherothrombotic plaque [80]. Type 2 MI has been identified as a potential etiology for heart injury during acute infection [80]. One aspect to consider is that the occurrence of cytokine storm leads to the catecholamines and secretion of IL-6, which subsequently elevate core body temperature, cardiac oxygen consumption, and heart rate.

By contrast, the presence of cytokine storm and endothelial dysfunction has an impact on the cardiac milieu, resulting in pathological alterations, such as thrombosis and coronary artery spasm. These pathological changes ultimately result in a reduction in blood supply through the coronary artery. The increase in heart rate caused by the reflex response results in a further reduction in myocardial perfusion due to the decrease in filling time. Insufficient oxygen delivery is exacerbated by severe hypotension, anemia, and hypoxemia in critically ill individuals afflicted with COVID-19. The convergence of these elements results in a discrepancy between the availability of oxygen and the body's requirement for it, resulting in the occurrence of acute heart injury.

Undoubtedly, when comparing type 1 MI resulting from plaque rupture and thrombus formation, it is evident that patients diagnosed with type 2 MI exhibit increased death rates. This disparity can be attributed, at least in part, to a greater prevalence of acute or long-lasting multimorbidity diseases among the population affected by type 2 MI [81]. Furthermore, Karakayalı et al. [82] documented that cardiovascular manifestations could both be at the micro- and macrovascular levels following a COVID-19 infection. Considering the advanced age and presence of comorbidities among hospitalized individuals with severe COVID-19, it is plausible to hypothesize that the occurrence of type 2 MI in these individuals may serve as a potential marker for the presence of more severe coronavirus infection and a poorer prognosis [15].

## Conclusions

The COVID-19 pandemic is often regarded as the most substantial public health problem of the current century. Due to its correlation with heightened mortality rates, CVD emerges as a prominent and significant comorbidity in the context of coronavirus and other respiratory viruses. Moreover, it stands as one of the primary contributors to mortality among those afflicted with COVID-19. The exact processes responsible for the cardiovascular symptoms observed in individuals with COVID-19 have yet to be fully understood and are likely to involve multiple factors. At present, the available information about direct cardiac virus toxicity is minimal. It is probable that both indirect and direct mechanisms work together to cause cardiovascular harm.

The COVID-19 pandemic has posed significant challenges to healthcare services and population well-being due to the implementation of quarantine restrictions. This is notably evident in the primary epidemic areas of coronavirus adnd in middle-income and low-income nations, such as South Africa, India, and Brazil. The potential consequence of this situation is the potential postponement of medical intervention for acute cardiovascular emergencies, such as stroke or AMI. Hence, it is imperative to enhance the level of consciousness and implement measures for self-protection in order to mitigate the incidence of morbidity and death among patients suffering from CVD and its associated ailments. Furthermore, telemedicine presents an additional potential avenue for healthcare accessibility in remote areas and facilitates regular follow-up for persons diagnosed with CVD. Currently, the available therapeutic interventions for COVID-19 are restricted to the administration of steroids, remedesivir, and potentially convalescent plasma. However, these medications have demonstrated suboptimal efficacy in terms of preventing infections, treating the disease, and mitigating its associated consequences.

It is imperative to closely monitor and treat the clinical cardiovascular manifestations linked to SARS-CoV-2 infection in order to mitigate the risk of mortality arising from cardiovascular problems. Simultaneously, it is imperative to prioritize intensive endeavors aimed at the development of a vaccination for COVID-19. It is worth noting that the administration of influenza vaccine has demonstrated a noteworthy reduction in the likelihood of cardiovascular events by 26-35% and a decrease in overall mortality by 43% among the adult population at large in the context of individuals at high risk who have experienced a recent AMI. The administration of influenza vaccine has demonstrated substantial advantages, as it resulted in a notable 55% reduction in the likelihood of cardiovascular events. Hence, it is imperative to prioritize COVID-19 immunization in individuals with CVD as a crucial measure for secondary prevention, particularly in those with cardiometabolic diseases, in order to mitigate the likelihood of heart-related events.
